# Exploring Symmetry-Independent Configurations in KTa_0.5_Nb_0.5_O_3_ Solid Solutions: A First-Principles, QTAIM, and AIMD Approach

**DOI:** 10.1021/acsomega.5c08252

**Published:** 2025-10-08

**Authors:** Jeronimo F. Silva, Ismael D. Souto, Julio Ricardo Sambrano, Anderson Reis Albuquerque, Ary S. Maia

**Affiliations:** † NPE-LACOM, 28097 Federal University of Paraíba, João Pessoa, PB 58051-900, Brazil; ‡ Modeling and Molecular Simulation Group, 153994 São Paulo State University, Bauru, SP 17033-360, Brazil; § Institute of Chemistry, 28123 Federal University of Rio Grande Do Norte, Natal, RN 59078-900, Brazil

## Abstract

Potassium tantaloniobate (KTN) is a lead-free perovskite solid solution with promising electro-optic, ferroelectric, and dielectric applications. Here, we present a systematic first-principles study of KTa_0.5_Nb_0.5_O_3_, exploring all 21 symmetry-independent configurations (SICs) in 2 × 2 × 2 cubic and tetragonal supercells. Configurational energy analysis identifies the most stable atomic arrangements, which are further characterized through band structure, density of states, and quantum theory of atoms in molecules (QTAIM) descriptors. Ab initio molecular dynamics (AIMD) simulations at finite temperature confirm the dynamical robustness of low-energy models and highlight subtle symmetry-dependent fluctuations. The results demonstrate how B-site ordering modulates electronic structure and bonding, offering guidance for the design of compositionally engineered perovskites for electro-optic and dielectric technologies.

## Graphical abstract

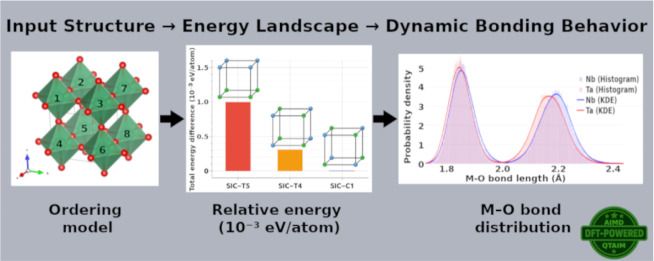

## 1 Introduction

Perovskite materials have attracted significant attention in materials science due to their wide range of applications, including solar cells,^1–3^ photocatalysis,^2,4^ light-emitting diodes,^5^ and lasers.^5^ These materials exhibit considerable structural diversity, encompassing 2D layered phases, 3D crystalline frameworks, hybrid organic–inorganic compounds, and double perovskites configured as solid solutions.^2,3,5^

A notable example within this class is potassium tantaloniobate Ta_0.5_Nb_0.5_O_3_, commonly referred to as KTN, a lead-free perovskite solid solution formed between KNbO_3_ and KTaO_3_. KTN displays a suite of functional properties, such as strong piezoelectric and pyroelectric responses, ferroelectric behavior, high dielectric permittivity, and prominent photoinduced effects.^6–10^

The complete miscibility of KNbO_3_ and KTaO_3_ arises from the chemical and structural similarities between Nb^5+^ and Ta^5+^ cations,^11^ enabling the precise tuning of material properties through controlled compositional variations^3,12^

Early studies by Triebwasser^13^ demonstrated that the KTa_*x*_Nb_(1‑*x*)_O_3_ solid solution undergoes phase transitions dependent on both temperature and composition. Specifically, the system adopts an orthorhombic phase at room temperature for *x* = 0, while for *x* ≈ 0.7 or under high-temperature conditions it transitions to a cubic structure. Notably, for *x* = 0.5, KTN crystallizes in the tetragonal space group under ambient conditions,^13^ a configuration of particular interest due to its exceptionally large electro-optic (EO) coefficient.^14–16^ As a result, KTN has long been considered a leading candidate for nonlinear optical applications.^10,17^

Despite being discovered over five decades ago, KTN continues to attract scientific interest. Only recently have high-quality KTN crystals, suitable in size and purity for optical applications, been successfully synthesized in the cubic phase.^15^

In addition, recent studies have highlighted new directions for KTN research. Ganesh et al. (2025)^18^ reported structural insights and demonstrated the biocompatibility of KTN, expanding its potential into biomedical applications. Similarly, Tong et al. (2025)^19^ achieved the hydrothermal growth of KTN crystals, underscoring progress in synthesis techniques and reinforcing the relevance of this material for both optical and multifunctional devices. These developments demonstrate that, although challenges remain in large-scale synthesis and stability control, KTN offers renewed opportunities for innovation across a broad range of technological fields.

To better understand the structure at *x* = 0.5, several research groups have explored possible atomic arrangements within the solid solution through theoretical investigations.,^17,20,21^ Yang et al.,^20^ for instance, proposed three high-symmetry models—[100]_NT_, [110]_NT_, and [111]_NT_—which refer to specific Ta/Nb ordering patterns within the supercell, characterized by the alternation of cations along the crystallographic directions [100], [110], and [111], respectively. These models were used to investigate the system’s elastic properties and mechanical stability. However, within a solid solution, many other atomic configurations beyond these idealized high-symmetry models are also possible. Surprisingly, the literature remains scarce in addressing such lower-symmetry variants. Moreover, there are inconsistencies among previous reports regarding both the relative stabilities of the common models and their associated space groups.

In this study, we performed a comprehensive computational evaluation of all possible symmetry-independent configurations (SICs) for both cubic and tetragonal phases of KTN, identifying the lowest-energy structures among them. These energetically favorable configurations were then analyzed in detail with respect to their structural, electronic, and thermodynamic properties using band structure and density of states calculations, simulated X-ray diffraction patterns, and harmonic vibrational analysis.

## 2 Methodology

### 2.1 Modeling and Computational Simulations

The calculations presented in this study were carried out using the CRYSTAL17 package,^22^ which employs atom-centered Gaussian basis functions to represent crystalline orbitals through linear combinations of Bloch functions. All computations were performed within the framework of Density Functional Theory (DFT),^23^ employing the B3LYP hybrid functional^24^ and incorporating Grimme’s D3 dispersion correction.^25^

The basis sets used were pob_TZVP_2012 for oxygen, SC_HAYWSC-31­(31d)­G for niobium, ECP60MDF-31­(51df)­G for tantalum, and SC_HAYWSC-31­(1d)­G for potassium, all of which are available in the CRYSTAL17 basis set library.^26^ The truncation thresholds for the Coulomb and exchange integrals were defined by the following five parameters: 10^–10^ (ITOL1), 10^–10^ (ITOL2), 10^–10^ (ITOL3), 10^–10^ (ITOL4), and 10^–20^ (ITOL5). k-point sampling was performed in the first Brillouin zone using the Monkhorst–Pack scheme,^27^ with a shrinking factor of 12 for the unit cell and 6 for the supercells. Geometry optimizations were fully converged using the following criteria: an energy convergence threshold (TOLDEE) of 2.721 × 10^–9^ eV/cell, a gradient threshold (TOLDEG) of 3.7024 × 10^–4^ eV/cell, and a displacement threshold (TOLDEX) of 1.481 × 10^–3^ eV/cell. All structures were fully optimized under these same conditions.

Two different unit cells were considered: a cubic structure, with lattice parameters obtained from the CIF file ICSD-190920 ^28^ (space group *Pm-3m*), and a tetragonal structure from ICSD-190921 ^28^ (space group *P4 mm*), both corresponding to KNbO_3_. After optimizing the unit cells, a 2 × 2 × 2 transformation matrix was applied to generate supercells (SCs), using periodic boundary conditions. These supercells were then fully reoptimized. Tests performed for larger supercell sizes confirmed a linear increase in total energy with system size, but at the expense of a considerably higher computational cost. For this reason, the systematic configurational analysis was restricted to the 2 × 2 × 2 supercell, which already captures all symmetry-independent configurations at *x* = 0.5. Although larger cells could in principle generate additional SICs, the main energetic trends are expected to be preserved. Each resulting structure contained eight niobium sites (Figure 1), of which half were replaced by tantalum atoms, forming the solid solution KNb_0.5_Ta_0.5_O_3_, which was subsequently subjected to a final geometry optimization.

**Figure 1:**
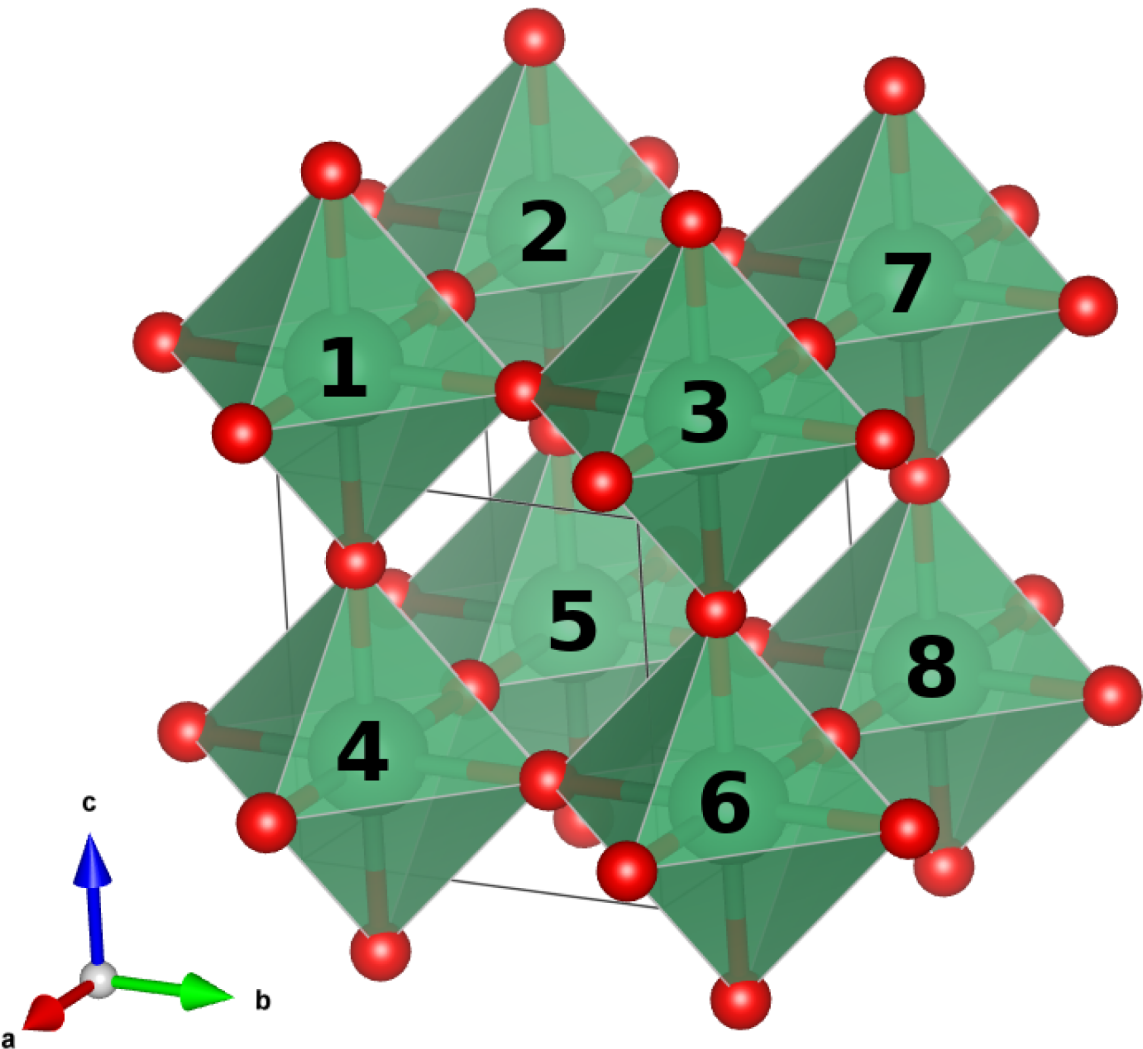
Distribution of the eight B-site positions in the 2 × 2 × 2 supercell. In the cubic lattice, all sites are symmetry-equivalent. In contrast, the tetragonal lattice differentiates between two types of positions, located in the top and bottom layers of the unit cell. The numbering reflects the site identification provided by CRYSTAL during execution.

Given the large number of distinct atomic arrangements within these supercells, the selection of substitution sites was guided by the identification of all Symmetry-Independent Configurations (SICs). This enumeration was carried out using the CONFCNT utility from the CRYSTAL17 package, which determines the number of configuration classes based on composition and site multiplicity, and provides one representative structure for each class.^29,30^ This approach enables a systematic sampling of the configurational space without redundancies.

Considering an atomic species *A* occupying an irreducible crystallographic position *d*, with multiplicity |*D*|, in a structure with symmetry group *G*. If another atomic species *X* replaces *A*, then |*D*| + 1 distinct compositions are possible, described by

1 $$A_{|D|-\alpha }X_{\alpha \;}\mathrm{where}\;\alpha =1...\left|D\right|$$

Under this condition, the number of different configurations—i.e., the distinct ways in which atoms *A* and *X* can be distributed over the |*D*| sites—is given by the binomial coefficient:

2 $$\left|S_{\alpha }\right|=\left(\begin{array}{c} \left|D\right|\\ \alpha \end{array}\right)=\frac{\left|D\right|!}{\alpha !\left(|D|-\alpha \right)!}$$

The total number of configurations across all compositions is therefore

3 $$\left|S\right|=2^{|D|}$$

The full set of configurations (*S*), when acted upon by the symmetry group *G*, is partitioned into |Δ­(*S*)| equivalence classes. Each class corresponds to a symmetry-independent configuration (SIC).

The thermodynamic stability of a set of distinct symmetry-independent configurations (SICs) in a crystalline system can be evaluated based on the energy differences between them.^31^ In the case of a solid solution composed of two chemical species, *A* and *B*, in proportions *x* and (1 - *x*) respectively, the configurational energy associated with a given SIC *i* can be expressed as

4 $$E_{i}^{\mathrm{conf}}=E_{(A_{1-x}B_{x}{)}_{i}}-\left[x\cdot E_{A}+\left(1-x\right)\cdot E_{B}\right]$$

In this expression, *E*(*A*_1–x_ *B*_x_)_i_ is the total energy of the system with composition *x*, corresponding to the *i* ^*th*^ SIC. The terms *E*_*A*_ and *E*_*B*_ represent the energies of the pure systems (i.e., when *x* = 1 and *x* = 0, respectively).

Thermodynamic evaluation of the system was performed using eq 4, with the composition fixed at *x* = 0.5, for each SIC derived from the studied supercells.

Electronic properties were investigated based on the optimized geometries corresponding to the thermodynamically most stable configurations. The band structures were calculated using a grid of 300 *k*-points along high-symmetry paths: path (Γ - *X* - *M* - Γ - *Z* - *R* - *A* - *Z*) for the tetragonal structure, and (Γ - *X* - *M* - Γ - *R* - *X*) for the cubic one.^32^

In order to further explore the electronic structure, a topological analysis of the electron density was performed within the framework of the Quantum Theory of Atoms in Molecules (QTAIM). This analysis was conducted using the TOPOND module^33^ implemented in the CRYSTAL17 package, and visualized with the TopIso3D Viewer,^34^ allowing for the identification of critical points and the evaluation of derived topological descriptors.

### 2.2 *Ab Initio* Molecular Dynamics

Ab initio molecular dynamics (AIMD) simulations were performed using the CP2K software package^35^ within the Born–Oppenheimer approximation and employing the GPW (Gaussian and Plane Waves) method. The electronic structure was calculated at the DFT level using the PBE functional with Grimme’s D3 dispersion correction. Goedecker-Teter-Hutter (GTH) pseudopotentials^36,37^ and MOLOPT basis sets^38^ were used for all elements. The wave function was expanded in a plane wave basis with a cutoff of 600 Ry and a relative cutoff of 50 Ry and the self-consistent field (SCF) convergence threshold was set to EPS_SCF = 1 × 10^–6^ a.u. A single Γ-point was used for Brillouin zone sampling. The simulations were carried out in the canonical ensemble (NVT) at 300 K, with temperature controlled using the CSVR thermostat^39^ (Canonical Sampling through Velocity Rescaling), employing a time constant of 8 fs. A time step of 1 fs was used for the integration of the equations of motion, and the trajectory comprised 5000 steps. The default self-consistent field (SCF) convergence criterion was set to 1 × 10^–14^ a.u. The simulation was conducted in the NVT ensemble, with fixed cell parameters, to isolate the effects of thermal motion on atomic positions while preserving the crystallographic symmetry and volume of the system. This choice is particularly suitable for systems where thermal expansion or pressure effects are not the primary focus. All AIMD simulations were performed under periodic boundary conditions, with the cell vectors held fixed. Prior to the production runs, each system was fully relaxed via geometry optimization at the same level of theory.

## 3 Results and Discussion

### 3.1 Configurational Analysis

By using the 2 × 2 × 2 supercell depicted in Figure 1, a 50% substitution was achieved through a Ta/Nb ratio of 4:4 across the eight *B*-sites. The exploration of configurations involving this partial substitution, as described in eq 1, resulted in 21 possible configurations, corresponding to the symmetry-independent classes (SICs) listed in Table 1, for both space groups considered. For each SIC, the table presents the specific substitution sites, the multiplicity of energetically equivalent configurations, and the cation ordering. A total of 6 SICs were identified for the cubic system, and 15 SICs for the tetragonal system.

**Table 1:** Symmetry-Independent Configurations (SICs) Resulting from 50% Ta Substitution in 2 × 2 × 2 Supercells

Ta/Nb ratio	Crystalline system	SIC	Subst. site	Multiplicity
4/4	Cubic	C1	2 3 4 8	2
		C2	4 5 6 8	6
		C3	2 3 5 6	6
		C4	1 2 3 4	8
		C5	2 3 6 7	24
		C6	1 2 3 8	24
	Tetragonal	T1	1 2 3 7	1
		T2	4 5 6 8	1
		T3	2 3 4 8	2
		T4	2 3 5 6	2
		T5	1 2 4 5	4
		T6	1 2 3 8	4
		T7	1 3 5 8	4
		T8	1 4 6 8	4
		T9	1 3 7 8	4
		T10	3 4 5 7	4
		T11	1 2 5 8	8
		T12	1 2 3 6	8
		T13	1 3 6 8	8
		T14	2 4 6 8	8
		T15	2 5 6 8	8

Previous studies have typically considered only three highly symmetric configurations, and exclusively within the tetragonal space group.^17,20^ However, restricting the analysis to these cases is insufficient to justify the preference for one configuration over another, especially considering that entropic effects may favor configurational disorder in solid solutions. Additionally, experimental studies have reported the occurrence of both cubic and tetragonal symmetries, depending on external conditions. In light of this, we systematically explored all possible configurations for both the cubic and tetragonal symmetries through single-point energy calculations performed individually for each SIC.

For the cubic system, the symmetry-independent configuration (SIC) with the lowest energy, SIC-C1, exhibited a rock-salt-like arrangement (Figure 2), in which each Nb atom is surrounded by six Ta atoms. This configuration corresponds to the [111]_NT_ model previously proposed by Yang et al.^20^ All other Ta-for-Nb substitutions in the cubic system resulted in higher total energies.

**Figure 2:**
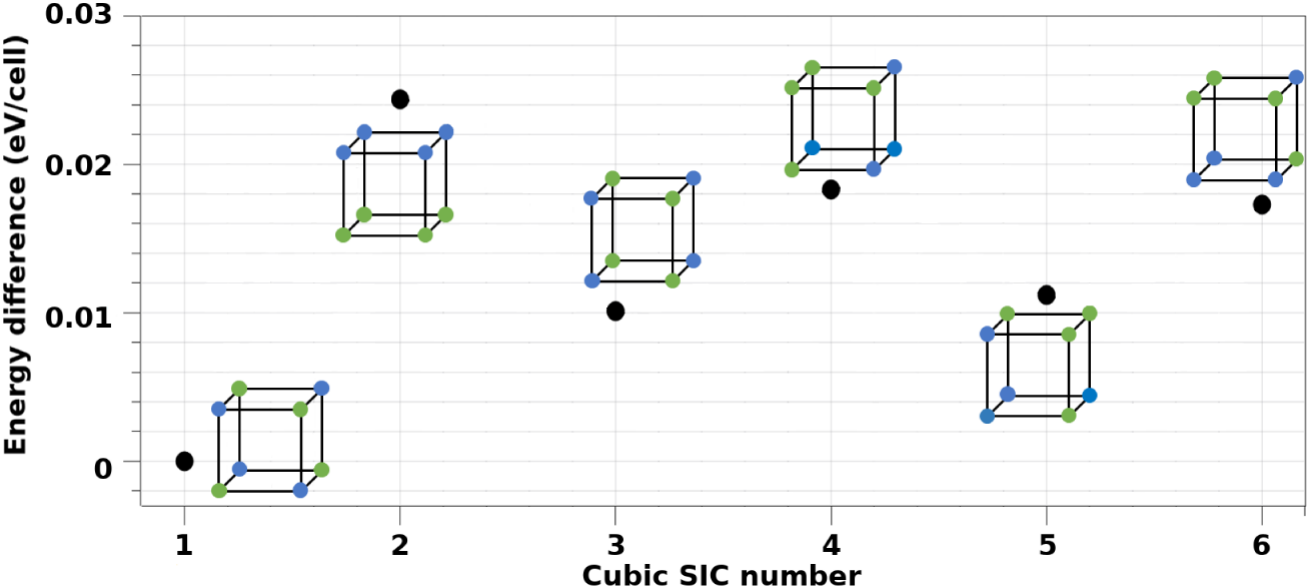
Relative energy differences (in eV/cell) among the six symmetry-independent configurations (SICs) identified for the cubic phase, referenced to the configuration with the lowest total energy.

The calculations performed for the 15 symmetry-independent configurations (SICs) of the tetragonal system revealed that SIC-T4 ([110]_NT_) is the most energetically stable configuration, as illustrated in Figure 3. Among all configurations, five stood out for their relatively low energies: SIC-T3, SIC-T4, SIC-T5, SIC-T12, and SIC-T15. Although some authors,^8,20,21^ have adopted SIC-T5 ([100]_NT_) as representative of the tetragonal phase due to its higher apparent symmetry, our results indicate that this configuration is not the most favorable in energetic terms. While the energy differences among the other four low-energy configurations are not substantial, the symmetry analysis reveals that SIC-T3 and SIC-T5 possess 4 and 2 symmetry operators, respectively, whereas SIC-T12 and SIC-T15 exhibit only one, reflecting a lower degree of structural symmetry in these configurations.

**Figure 3:**
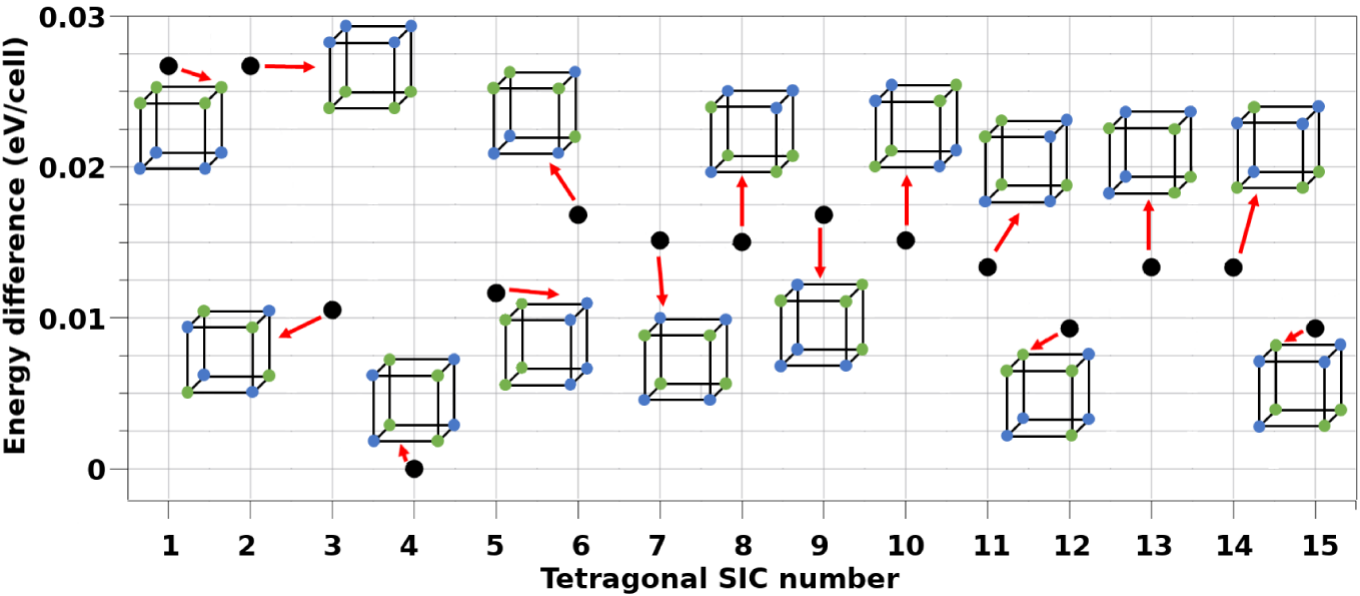
Relative energy differences (in eV/cell) among the 15 symmetry-independent configurations (SICs) identified for the tetragonal phase, referenced to the configuration with the lowest total energy.

For a comparative assessment, three high-symmetry configurations were selected from the 21 SICs: SIC-T5 ([100]_NT_), SIC-T4 ([110]_NT_), and SIC-C1 ([111]_NT_). These configurations are summarized in Table 2 and were further examined in terms of energetic and structural descriptors. Due to symmetry breaking introduced by Ta/Nb substitution, new sets of symmetry operators were identified using the FINDSYM tool,^40^ and the corresponding crystallographic information files (CIFs), generated from the optimized geometries, are provided in the Supporting Information.

**Table 2:** Comparison of Lattice Parameters (*a*, *b*, *c*), Band Gaps (*E*_gap_), and Assigned Space Groups for Selected Models in This Work and Previous Studies

This work (DFT/B3LYP-D3)					
Model	*a* (Å)	*b* (Å)	*c* (Å)	*E*_gap_ (eV)	Space group
SIC-T5	3.975	3.975	3.992	3.25	*Cm* (*P4/mmm*)
SIC-T4	3.974	3.974	3.996	3.27	*Amm2*
SIC-C1	3.978	3.978	3.978	3.27	*R3m* (*Fm*3̅*m*)
**Shen and Zhou** ^17^ **(DFT/GGA)**					
[100]	4.029	4.029	4.115	1.385	*P4 mm*
[110]	4.058	4.058	4.033	1.858	*Pmm2*
[111]	4.052	4.052	4.051	2.020	*R3m*
**Yang et al.** ^20^ **(DFT/LDA-PZ)**					
[100]	3.998	3.998	3.987	–	*P4/mmm*
[110]	3.989	3.989	3.999	–	*P4/mmm*
[111]	3.991	3.991	3.991	–	*Fm*3̅*m*
**Zhang et al.** ^21^ **(DFT/GGA)**					
[100]	4.059	4.060	4.060	1.58	*P4/mmm*
[110]	5.742	5.742	4.060	1.87	*P4/mmm*
[111]	4.060	4.060	4.060	1.76	*Fm*3̅*m*

Figure 4 illustrates the total energy differences per atom among these three selected configurations. Although the energy differences are small (within 1.5 meV/atom), a subtle stability trend is observed, with the [111]_NT_ model being the most stable, followed by [110]_NT_, and finally [100]_NT_. The small magnitude of these energy gaps highlights the near-degeneracy of configurations in KTN, reinforcing the complexity of capturing stability based solely on static energy criteria. Nonetheless, this trend will be reevaluated in light of the structural and dynamic behavior revealed by AIMD simulations in the subsequent sections.

**Figure 4:**
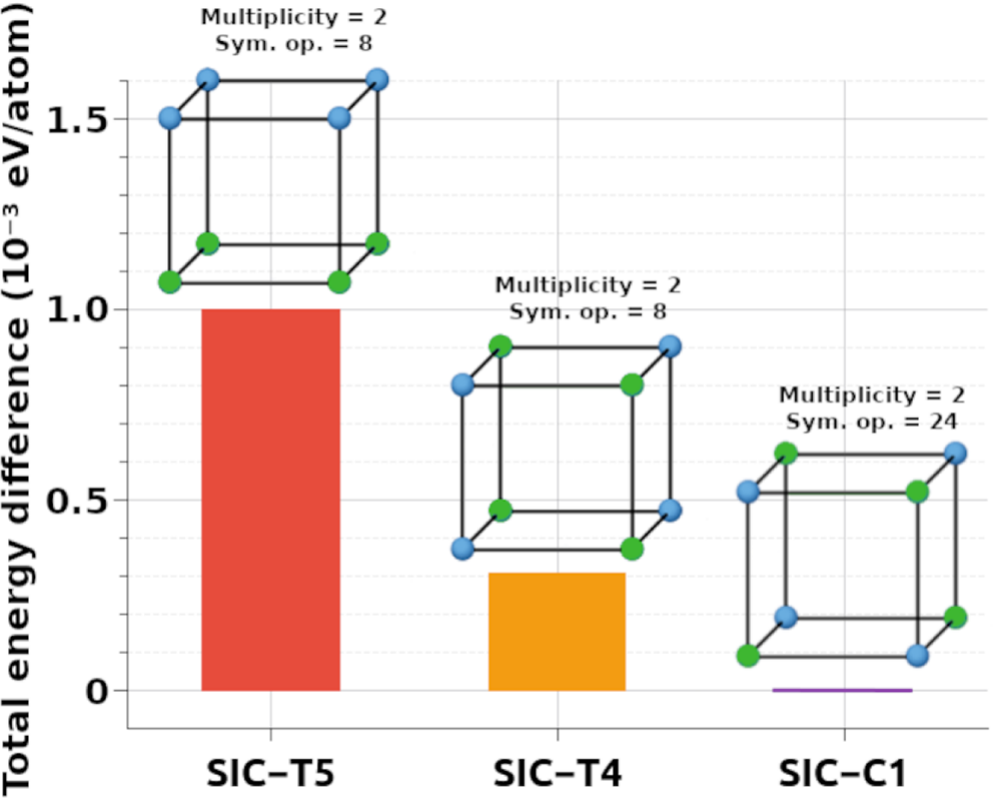
Total energy difference per atom for the three structural models (SIC-T5, SIC-T4, and SIC-C1), referenced to the most stable configuration (SIC-C1). The schematic polyhedra illustrate the atomic arrangements used, along with their corresponding symmetry operations and multiplicities.

For the SIC-C1 model, the symmetry analysis identified the rhombohedral space group *R3m*, in agreement with the results reported by Shen and Zhou.^17^ However, when adopting lower precision criteria for the identification of symmetry operators, it becomes possible to attribute a higher-symmetry classification. Under such conditions, our results align with those of Yang et al.^20^ and Zhang et al.^21^ who reported the cubic space group *Fm*3̅*m*.

In the case of SIC-T4, the space group determined was *Amm2*, which is consistent with the findings of Di Geronimo et al.^41^ In their study of KTN solid solutions with Ta/Nb ratios of 40/60, 50/50, and 60/40, they noted that although the 50/50 composition is often described as tetragonal, it is more accurately characterized by the orthorhombic space group *Amm2*.

The symmetry classification of SIC-T5 also showed sensitivity to the precision level of the symmetry analysis. Depending on the parameters adopted, the structure may be assigned to either the monoclinic space group *Cm* or the tetragonal space group *P4/mmm*, as reported by Yang et al.^20^ and Zhang et al.^21^

A comparison of the calculated lattice parameters and band gaps for the selected models is presented in Table 2. The tetragonal configurations SIC-T4 and SIC-T5 exhibit slight anisotropy, with lattice constants satisfying *c* > *a* = *b*, consistent with the expected distortion in noncubic perovskite supercells. In contrast, the SIC-C1 model presents a fully cubic unit cell (*a* = *b* = *c* = 3.978 Å). Regarding the electronic structure, all three models exhibit direct band gaps at the Γ point, with values in the range of 3.25–3.27 eV. These values are noticeably larger than those reported in previous GGA-based studies,^17,20,21^ as anticipated from the use of the B3LYP hybrid functional. Experimental measurements report a band gap of 3.25 eV for KTa_0.5_Nb_0.5_O_3_,^4^ in excellent agreement with our hybrid-DFT results, acknowledging the expected differences between theoretical and experimental estimates. Altogether, these results indicate that variations in local symmetry lead to subtle structural distortions that, in turn, affect orbital hybridization and the resulting electronic properties within the solid solution.

### 3.2 Electronic Evaluation

Figure 5 presents the electronic band structures and projected density of states (DOS) for the three investigated configurations: SIC-T5 (a), SIC-T4 (b), e SIC-C1 (c). All models exhibit a direct band gap at the Γ point, with values ranging from 3.25 to 3.27 eV. This confirms that, despite structural variations, the optical response of the system is expected to be direct in nature and dominated by transitions at the Brillouin zone center.

**Figure 5:**
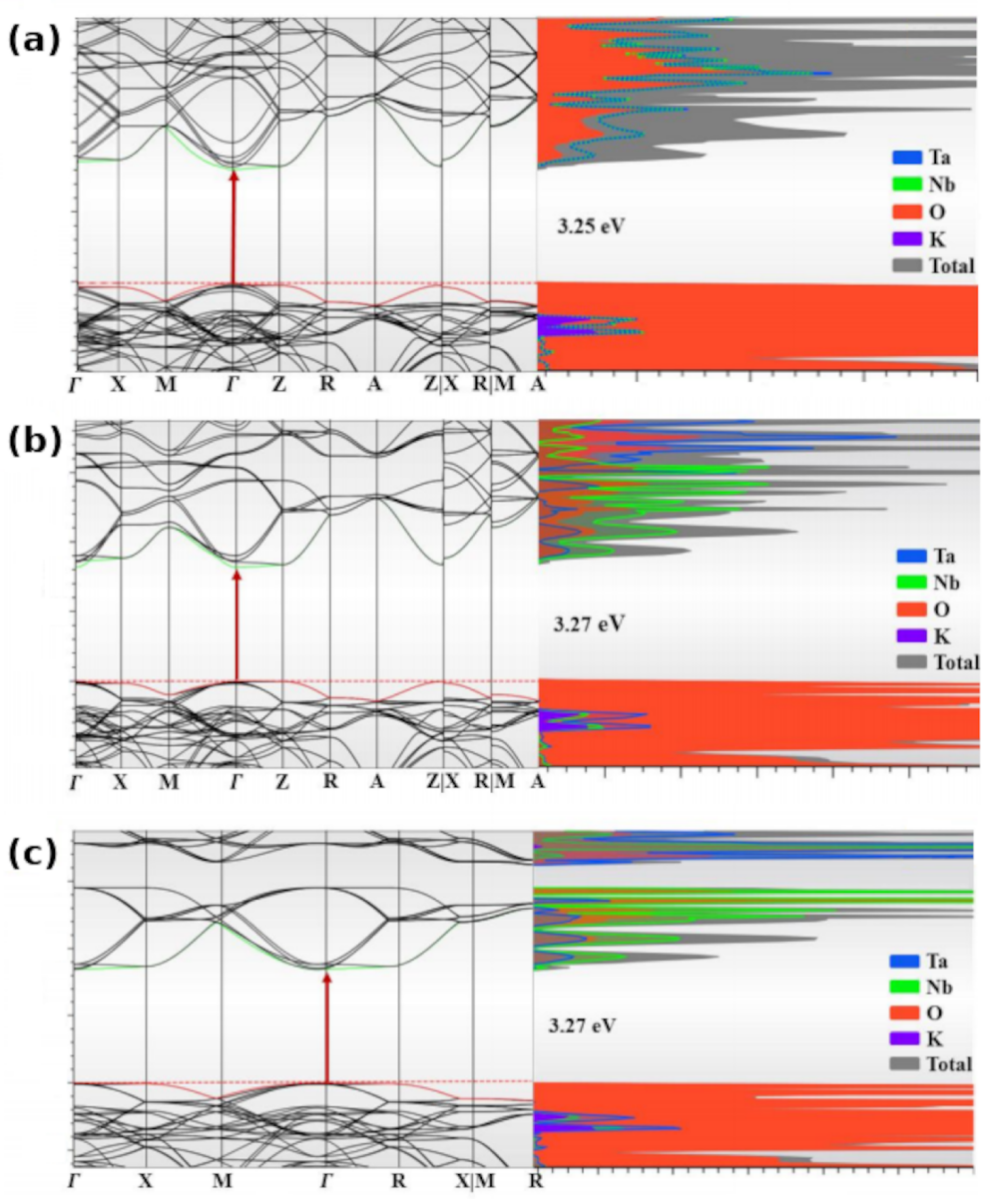
Electronic band structures and projected density of states (DOS) for (a) SIC-T5 ([[100]_NT_), (b) SIC-T4 ([110]_NT_), and (c) SIC-C1 ([111]_NT_). Band structure paths follow the high-symmetry k-point trajectory proposed by Setyawan and Curtarolo.^32^

While the general shape of the band structures remains consistent across the three models, subtle but significant differences arise in the orbital composition of the conduction band minimum. In particular, the contribution of oxygen states decreases in the following order: SIC-T5 > SIC-C1 > SIC-T4. This trend suggests that the [100]­NT configuration (SIC-T5) promotes stronger hybridization between O 2p orbitals and B-site cations (Nb/Ta), which may enhance the covalent character of the B–O bonds. Such hybridization can influence not only transition probabilities and carrier effective masses, but also polaron formation and photoresponse properties. In contrast, the lower oxygen participation observed in SIC-T4 indicates a more ionic character in the conduction band, potentially affecting carrier localization and dielectric behavior. These distinctions highlight the importance of cation ordering in modulating the electronic and optical properties of KTN solid solutions.

### 3.3 Topological Evaluation

To further elucidate the nature of chemical bonding in the three KTN configurations, we examined the relationship between the Bond Degree (*H*/ρ) and the dimensionless ratio (|*V*|/*G*) for each bond critical point (BCP), as shown in Figure 6. Here, ρ is the electron density at the BCP, *G* is the kinetic energy density, *V* is the potential energy density, and *H* is the total energy density defined as *H* = *G* + *V*. The bond degree descriptor (*H*/ρ) provides insight into the covalent versus ionic character of the interaction, while the ratio (|*V*|/*G*) serves as an indicator of bond strength and localization. This correlation plot distinctly separates the three bond types (Nb–O, Ta–O, and K–O), revealing two well-defined clusters: a high bond degree region associated with metal–oxygen bonds (Nb–O and Ta–O), and a low bond degree region corresponding to K–O interactions. Within the metal–oxygen cluster, a clear trend emerges where increasing bond degree correlates with increasingly negative dimensionless ratios, indicating greater electron density concentration and thus stronger bonding interactions. Notably, Nb–O and Ta–O bonds occupy similar ranges of bond descriptors, but exhibit subtle differences across the three structural models. For instance, in the [111] structure, the O–Ta interactions exhibit the highest bond degree and most negative dimensionless ratio, suggesting more localized and directional bonding in this configuration. These results reinforce the partial covalent nature of the Nb–O and Ta–O bonds, while the K–O interactions remain weak and primarily ionic. This trend reflects changes in the balance between potential and kinetic energy densities, and highlights the influence of structural symmetry on bond character and localization.

**Figure 6:**
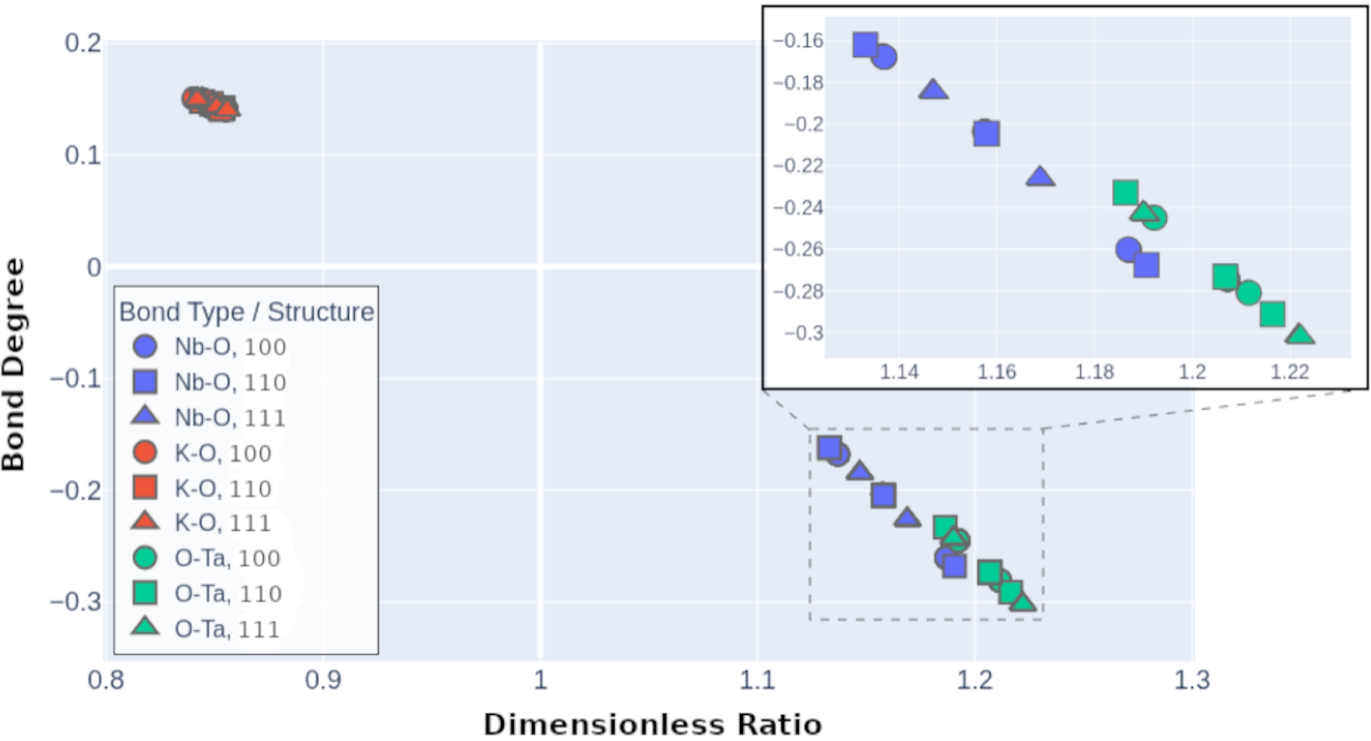
Correlation between the bond degree (*H*/ρ) and the dimensionless ratio (|*V*|/*G*) for bond critical points (BCPs) in the KTN models ([100]_NT_, [110]_NT_, and [111]_NT_). Each point represents a BCP, classified by bond type (Nb–O, Ta–O, or K–O). Weaker K–O interactions appear in the upper left, while stronger B–O bonds cluster in the lower right. The inset highlights subtle increases in |*V*|/*G* for B–O bonds in the [111]_NT_ model, especially for Ta–O.

Complementing the analysis of Figure 6, the boxplot shown in Figure 7 presents the distribution of electron density (ρ) at the bond critical points (BCPs) for the three model structures. A clear separation is observed between the Nb–O/Ta-O bonds and the more ionic K–O bonds, the latter exhibiting significantly lower electron density values, as expected. Among the Nb–O interactions, the [100]_NT_ structure displays a broader distribution of ρ, ranging from 0.09 to 0.14 au, suggesting greater variability in the local bonding environments. In contrast, the [111]_NT_ and [110]_NT_ models present narrower distributions, centered around similar mean values. For the Ta–O bonds, the [111]_NT_ structure exhibits the highest average ρ, reinforcing its comparatively stronger Ta–O interactions. These results support the interpretation provided by the Dimensionless Ratio and Bond Degree analysis, further confirming the chemical differentiation between the B-sites and their local coordination across the three models.^34,42^

**Figure 7:**
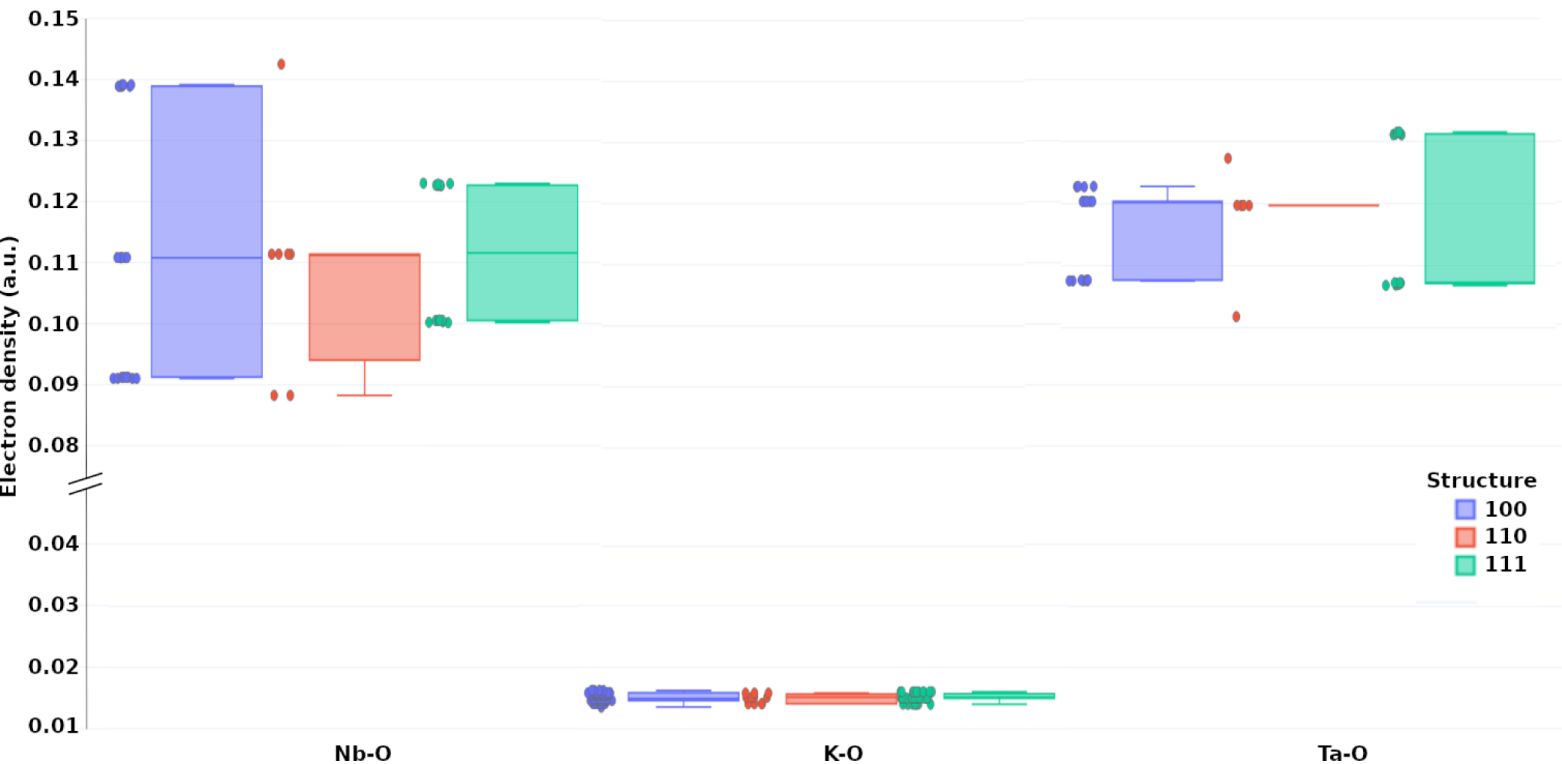
Boxplot of electron density (ρ) at BCPs for the three KTN models ([100]_NT_, [110]_NT_, and [111]_NT_), grouped by bond type (K–O, Nb–O, and Ta–O). Individual data points are also shown alongside each boxplot to highlight the distribution within each group. K–O bonds show lower ρ, reflecting their ionic nature, while B–O bonds exhibit higher values. The [111]_NT_ model shows slightly higher ρ for Ta–O, and the broader Nb–O distribution in [100]_NT_ suggests greater local variability. These trends complement those observed in the |*V*|/*G* vs *H*/ρ analysis.

### 3.4 AIMD Evaluation

To investigate the dynamic and structural behavior of the KTN solid solution at finite temperature, *ab initio* molecular dynamics (AIMD) simulations were performed at 300 K for the three representative configurations [100]_NT_, [110]_NT_, and [111]_NT_. All simulations were conducted in the canonical (NVT) ensemble, maintaining fixed lattice parameters to ensure structural comparability and suppress unphysical cell distortions that may arise in small supercells under variable pressure conditions. This approach is particularly appropriate for KTN, a perovskite with low compressibility and moderate thermal expansion, where volume fluctuations are expected to be minimal. The AIMD trajectories, each extending over 5 ps, were analyzed using a combination of structural descriptors, including root-mean-square displacements (RMSD) and M-O bond distance distributions, to evaluate the degree of dynamic disorder and structural stability exhibited by each model.

Figure SI-1 shows the probability density distributions of M-O bond lengths for the KTN models [100]_NT_, [110]_NT_, and [111]_NT_. Although a clear bimodal distribution of bond lengths is observed—reflecting structural features inherent to the octahedral environment—the high degree of similarity among the three configurations makes it difficult to discern any meaningful trend throughout the AIMD trajectory. In contrast, the standard deviation of M-O bond lengths within individual octahedra, computed at each frame along the trajectory (Figure 8), provides a clearer view of the instantaneous asymmetry of the coordination environment. This approach reveals how local distortions evolve over time across the different structural models.

**Figure 8:**
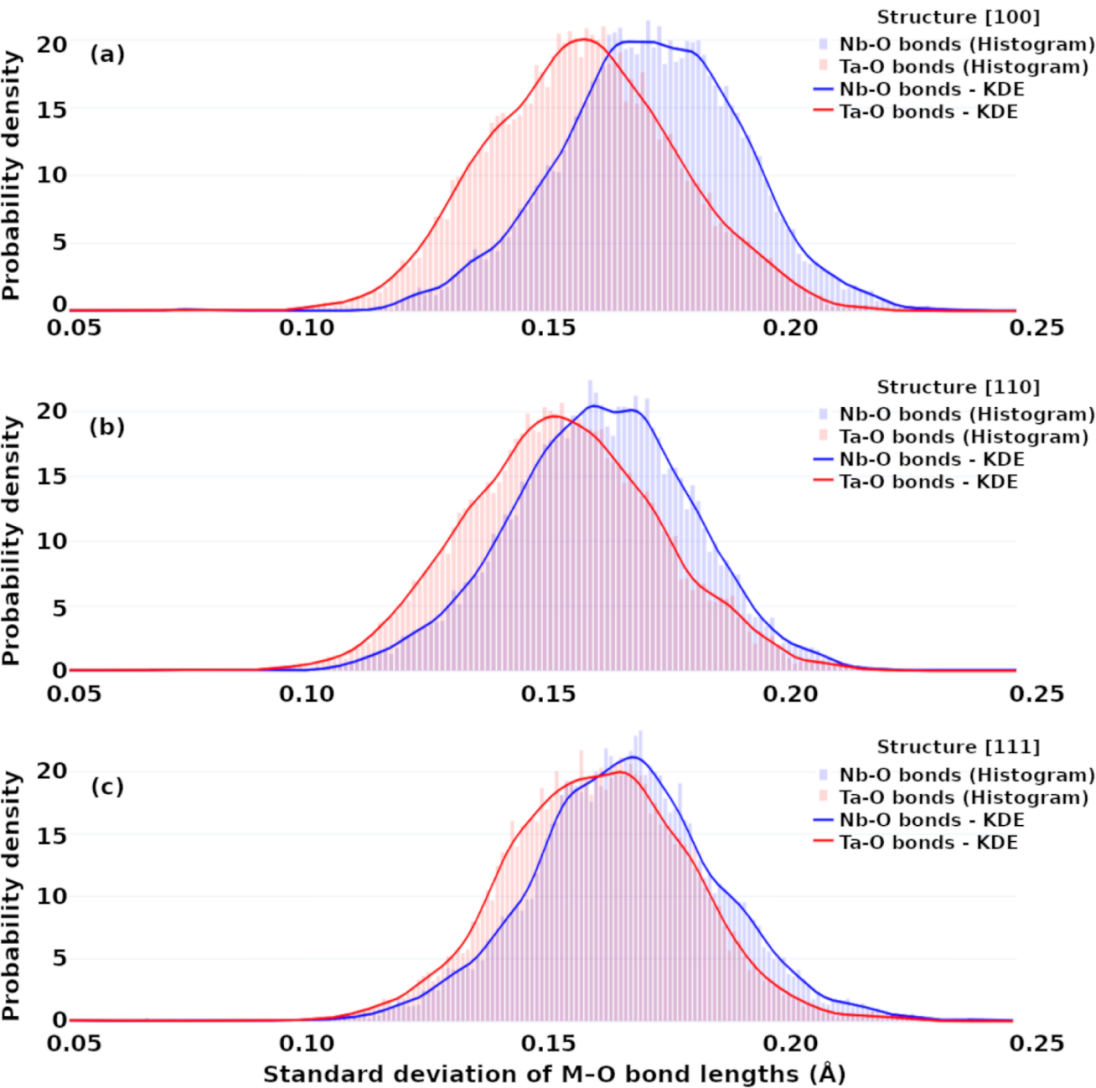
Probability density distribution of the standard deviation of M-O bond lengths (Nb–O and Ta–O) per octahedron in KTN supercells with B-site cation arrangements along (a) [100]_NT_, (b) [110]_NT_, and (c) [111]_NT_ directions. Histograms represent the raw data, while the solid lines correspond to kernel density estimates (KDE) for each element.

Although the distribution of M-O distances does not clearly differentiate the models in terms of structural stability, the standard deviation analysis reveals a compelling trend. The [111]_NT_ model exhibits a more homogeneous and narrow distribution for both Nb–O and Ta–O environments, suggesting reduced local anisotropy and enhanced dynamic uniformity.

Figure 9 presents the RMSD evolution over time for the three structural models—[100]_NT_, [110]_NT_, and [111]_NT_, alongside the results of the clustering analysis applied to the RMSD profiles.

**Figure 9:**
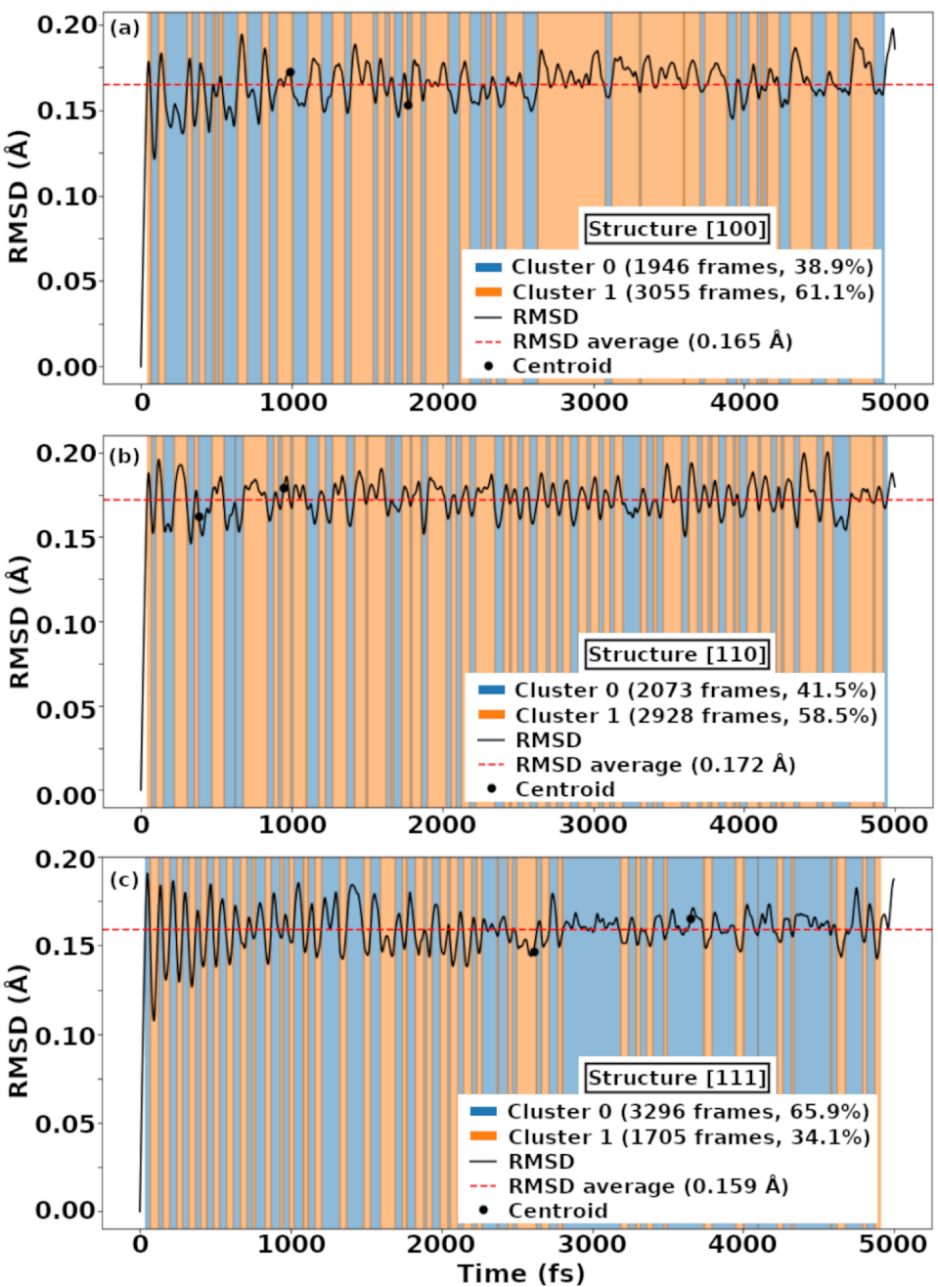
Figure 9: Root mean square deviation (RMSD) along the AIMD trajectories for the three KTN configurations: (a) [100]_NT_, (b) [110]_NT_, and (c) [111]_NT_. The background colors indicate cluster assignments obtained via KMeans clustering (k = 2), with the number and percentage of frames in each cluster shown in the legend. The black line represents the RMSD curve, the red dashed line indicates the average RMSD, and the black dots mark the centroids of each cluster.

The [110]_NT_ configuration displays frequent transitions between clusters, indicative of a higher degree of structural fluctuation during the AIMD trajectory. In contrast, the [111]_NT_ model remains predominantly in a single cluster, while the [100]_NT_ structure exhibits intermediate behavior, with longer periods of stability interspersed with moderate transitions. The trend in average RMSD values across the three configurations—[111]_NT_ (0.159 Å) < [100]_NT_ (0.165 Å) < [110]_NT_ (0.172 Å)—is consistent with the clustering results, supporting the interpretation that the [111]_NT_ model exhibits greater configurational persistence. Although the RMSD differences are subtle, they reveal a clear hierarchy in structural stability under thermal conditions, with the [111]_NT_ configuration emerging as the most structurally resilient.

This trend partially aligns with the total energy calculations (Figure 4) and the QTAIM-based topological analysis (Figures 6 and 7), both of which also favor the [111]_NT_ configuration. However, it is important to note that energetic and structural descriptors capture distinct aspects of stability, and the small energy differences between models suggest that all configurations are relatively close in thermodynamic viability.

## 4 Conclusion

In this study, we conducted a comprehensive first-principles investigation of the KTa_0.5_Nb_0.5_O_3_ (KTN) solid solution, evaluating its thermodynamic, structural, electronic, and dynamic properties across multiple symmetry-independent configurations (SICs). Among the 21 SICs examined, the [111]_NT_ model emerged as the most energetically stable for the cubic phase, while [110]_NT_ exhibited the lowest energy in the tetragonal system. Despite the small energy differences between these configurations, our analysis revealed subtle but consistent trends favoring the [111]_NT_ model.

The topological analysis based on QTAIM descriptors confirmed the mixed transient-covalent nature of the Nb–O and Ta–O bonds, with Ta–O interactions showing slightly higher bond degrees and electron densities at the bond critical points—suggesting a marginally more covalent character. This trend was particularly evident in the [111]_NT_ configuration, which exhibited stronger and more localized metal–oxygen bonding.

AIMD simulations further revealed that the [111]_NT_ model maintained a more uniform structural behavior at 300 K, as evidenced by the narrower distributions of M-O bond length deviations and a lower average RMSD. The clustering analysis of the AIMD trajectories corroborated this result, showing that the [111]_NT_ structure remains predominantly within a single dynamic regime throughout the simulation, indicating enhanced configurational persistence.

Although the RMSD-based structural stability and the QTAIM bonding features support the superior resilience of the [111]_NT_ configuration, the total energy differences between the models are relatively small. This highlights the delicate balance between energetic and structural criteria in assessing the stability of solid solutions. Overall, our findings provide a robust computational framework for understanding cation ordering effects in KTN and similar perovskite systems, offering valuable insights for their design and application in optical and dielectric devices.

The present analysis was conducted using a 2 × 2 × 2 supercell, which captures all symmetry-independent configurations at *x* = 0.5. Larger supercells would naturally allow additional cation arrangements and could further refine the energetic hierarchy. However, given the small energy differences observed among the lowest-energy states, the main stability trends (favoring [111] ordering) are expected to persist at larger scales. Since all 21 CIFs of the 2 × 2 × 2 supercell were explicitly considered, a canonical ensemble analysis could in principle be performed to estimate thermodynamic populations, which may be explored in future work.

## Supplementary Material



## Notes

Calculated crystallographic information files, probability density distributions of M-O bond lengths (*M* = Nb or Ta) obtained from ab initio molecular dynamics (AIMD) simulations (PDF)
