# Supplementary material for: Exploring Symmetry-Independent Configurations in KTa0.5Nb0.5O3 Solid Solutions: A First-Principles, QTAIM, and AIMD Approach
Source: ACS Omega. 2025 Oct 8;10(41):49158–67. doi: 10.1021/acsomega.5c08252 (PMC12547805; doi:10.1021/acsomega.5c08252)
Supplement: Supplementary file 1 [file ao5c08252_si_001.pdf]

SUPPORTING INFORMATION

Exploring Symmetry-Independent Configurations in $KTa_{0.5}Nb_{0.5}O_3$ Solid Solutions: A First-Principles, QTAIM, and AIMD Approach

Jeronimo F. Silva,[†] Ismael D. Souto,[†] Julio Ricardo Sambrano ,[‡] Anderson Reis Albuquerque,[¶] and Ary S. Maia[*,†]

†*NPE-LACOM, Federal University of Paraíba, João Pessoa, PB, 58051-900, Brazil*

‡*Modeling and Molecular Simulation Group, São Paulo State University, Bauru, SP, 17033-360, Brazil*

¶*Institute of Chemistry, Federal University of Rio Grande do Norte, Natal, RN, 59078-900, Brazil*

E-mail: arymaia@quimica.ufpb.br

Phone: +55(83)32167441

1 Calculated CIFs

1.1 SIC-T5 ($[100]_{NT}$)

```
################################################################
#
#                    CRYSTAL17 CIF FILE
#
################################################################

data_OPT_STEP_0

_symmetry_space_group_name_H-M 'P 1'
_cell_length_a      7.97477231
_cell_length_b      7.97416933
_cell_length_c      7.94462160
_cell_angle_alpha   90.001334
_cell_angle_beta    89.998526
_cell_angle_gamma   90.071481

loop_
_atom_site_label
_atom_site_fract_x
_atom_site_fract_y
_atom_site_fract_z
K1    3.463331964601E-03  2.213399993291E-04 -4.613119380074E-03
K2    3.457463519548E-03  2.230903253052E-04  4.944946095668E-01
```

K3	3.460974779757E-03	-4.997779539395E-01	-4.615272543095E-03
K4	3.456947304303E-03	-4.997750580376E-01	4.944940555090E-01
K5	-4.965377553300E-01	2.253663042853E-04	-4.616962613390E-03
K6	-4.965433035142E-01	2.225246790644E-04	4.944948651290E-01
K7	-4.965390537240E-01	-4.997771015387E-01	-4.616270975875E-03
K8	-4.965415825719E-01	-4.997726133364E-01	4.944932019662E-01
NB9	2.466860568879E-01	2.569511320356E-01	-2.550761691578E-01
TA10	2.533171567995E-01	2.503562752792E-01	2.449291829126E-01
NB11	2.466906192741E-01	-2.430494410582E-01	-2.550773657690E-01
TA12	2.533157925772E-01	-2.496433882309E-01	2.449277851076E-01
NB13	-2.533149317558E-01	2.569515576285E-01	-2.550735622596E-01
TA14	-2.466832984698E-01	2.503553779766E-01	2.449314866594E-01
NB15	-2.533081909007E-01	-2.430471198606E-01	-2.550775229811E-01
TA16	-2.466838344316E-01	-2.496442598641E-01	2.449281235755E-01
O17	2.580737233288E-01	2.456710594404E-01	-3.431143226384E-03
O18	2.580538752125E-01	2.456757238363E-01	4.932876795179E-01
O19	2.580723031871E-01	-2.543341100629E-01	-3.430917518677E-03
O20	2.580543208460E-01	-2.543215152717E-01	4.932880942795E-01
O21	-2.419325075695E-01	2.456679075263E-01	-3.429627574053E-03
O22	-2.419416981771E-01	2.456763454979E-01	4.932890266546E-01
O23	-2.419299344252E-01	-2.543317426103E-01	-3.431204366177E-03
O24	-2.419416186403E-01	-2.543232216221E-01	4.932877848702E-01
O25	6.786971254857E-03	2.473877436374E-01	2.449508034826E-01
O26	7.903786130860E-03	2.439892946783E-01	-2.550341177570E-01
O27	6.787121814250E-03	-2.526111605345E-01	2.449512882989E-01
O28	7.903517712785E-03	-2.560099122843E-01	-2.550371251878E-01
O29	-4.932133032758E-01	2.473872231301E-01	2.449552957505E-01

```
O30  -4.920976377702E-01  2.439882987286E-01 -2.550398224300E-01
O31  -4.932125201839E-01 -2.526114281697E-01  2.449543573365E-01
O32  -4.920948237141E-01 -2.560099715233E-01 -2.550408958000E-01
O33   2.563096758447E-01 -3.073500367136E-03  2.449006967919E-01
O34   2.597895512359E-01 -4.165326201113E-03 -2.551061497222E-01
O35   2.563060329667E-01  4.969267453549E-01  2.449072203209E-01
O36   2.597893115679E-01  4.958357733558E-01 -2.551108673083E-01
O37  -2.436912187124E-01 -3.073725720651E-03  2.449033625213E-01
O38  -2.402097414079E-01 -4.164164485331E-03 -2.551079271112E-01
O39  -2.436937659995E-01  4.969261916154E-01  2.449055656795E-01
O40  -2.402110726484E-01  4.958341662483E-01 -2.551084422484E-01

#===END
```

1.2 SIC-T4 ($[110]_{NT}$)

```
################################################################
#
#                    CRYSTAL17 CIF FILE
#
################################################################

data_OPT_STEP_0

_symmetry_space_group_name_H-M 'P 1'
_cell_length_a      7.94733660
_cell_length_b      7.94733660
_cell_length_c      7.99206802
_cell_angle_alpha   90.000000
_cell_angle_beta    90.000000
_cell_angle_gamma   89.999954

loop_
_atom_site_label
_atom_site_fract_x
_atom_site_fract_y
_atom_site_fract_z
K1    0.000000000000E+00  0.000000000000E+00 -2.590292093909E-03
K2    0.000000000000E+00  0.000000000000E+00  4.974103519479E-01
K3    0.000000000000E+00 -5.000000000000E-01 -2.590923810129E-03
K4    0.000000000000E+00 -5.000000000000E-01  4.974095099582E-01
```

K5	-5.000000000000E-01	-1.058791184068E-22	-2.590923810129E-03
K6	-5.000000000000E-01	-1.058791184068E-22	4.974095099582E-01
K7	-5.000000000000E-01	-5.000000000000E-01	-2.588883177473E-03
K8	-5.000000000000E-01	-5.000000000000E-01	4.974117068595E-01
TA9	2.499930965247E-01	2.499930965247E-01	-2.518102320359E-01
TA10	2.499929726991E-01	2.499929726991E-01	2.481897050250E-01
NB11	2.500073816674E-01	-2.500073816674E-01	-2.449830697523E-01
NB12	2.500074965480E-01	-2.500074965480E-01	2.550170053545E-01
NB13	-2.500073816674E-01	2.500073816674E-01	-2.449830697523E-01
NB14	-2.500074965480E-01	2.500074965480E-01	2.550170053545E-01
TA15	-2.499930965247E-01	-2.499930965247E-01	-2.518102320359E-01
TA16	-2.499929726991E-01	-2.499929726991E-01	2.481897050250E-01
O17	2.499997415500E-01	2.499997415500E-01	-7.722586389322E-03
O18	2.499996703677E-01	2.499996703677E-01	4.922773955239E-01
O19	2.500014787497E-01	-2.500014787497E-01	-7.612572418432E-03
O20	2.500015558186E-01	-2.500015558186E-01	4.923876185271E-01
O21	-2.500014787497E-01	2.500014787497E-01	-7.612572418432E-03
O22	-2.500015558186E-01	2.500015558186E-01	4.923876185271E-01
O23	-2.499997415500E-01	-2.499997415500E-01	-7.722586389322E-03
O24	-2.499996703677E-01	-2.499996703677E-01	4.922773955239E-01
O25	1.425073515265E-03	2.500010391097E-01	2.416520686711E-01
O26	1.425129698221E-03	2.500010814556E-01	-2.583473123416E-01
O27	-1.425073515265E-03	-2.500010391097E-01	2.416520686711E-01
O28	-1.425129698221E-03	-2.500010814556E-01	-2.583473123416E-01
O29	4.985738052419E-01	2.500009021210E-01	2.416514639415E-01
O30	4.985737702608E-01	2.500009303063E-01	-2.583478661467E-01
O31	-4.985738052419E-01	-2.500009021210E-01	2.416514639415E-01

```
O32  -4.985737702608E-01 -2.500009303063E-01 -2.583478661467E-01

O33   2.500010391097E-01  1.425073515265E-03  2.416520686711E-01

O34   2.500010814556E-01  1.425129698221E-03 -2.583473123416E-01

O35   2.500009021210E-01  4.985738052419E-01  2.416514639415E-01

O36   2.500009303063E-01  4.985737702608E-01 -2.583478661467E-01

O37  -2.500010391097E-01 -1.425073515265E-03  2.416520686711E-01

O38  -2.500010814556E-01 -1.425129698221E-03 -2.583473123416E-01

O39  -2.500009021210E-01 -4.985738052419E-01  2.416514639415E-01

O40  -2.500009303063E-01 -4.985737702608E-01 -2.583478661467E-01


#===END
```

1.3 SIC-C1 ($[111]_{\mathrm{NT}}$)

```
#################################################################
#
#                    CRYSTAL17 CIF FILE
#
#################################################################

data_OPT_STEP_0

_symmetry_space_group_name_H-M 'P 1'
_cell_length_a      7.96166987
_cell_length_b      7.96094750
_cell_length_c      7.96094750
_cell_angle_alpha   89.977299
_cell_angle_beta    89.976562
_cell_angle_gamma   89.976562

loop_
_atom_site_label
_atom_site_fract_x
_atom_site_fract_y
_atom_site_fract_z
K1    3.149237778193E-04 -9.089575595042E-04 -9.089575595042E-04
K2    3.472721298840E-04 -8.757623933620E-04  4.991185689404E-01
K3    3.472721298840E-04  4.991185689404E-01 -8.757623933620E-04
K4    3.087833939994E-04  4.990823900559E-01  4.990823900558E-01
```

K5	-4.996520519633E-01	-8.776693779938E-04	-8.776693779938E-04
K6	-4.996879176573E-01	-9.093410774992E-04	4.990864877013E-01
K7	-4.996879176573E-01	4.990864877013E-01	-9.093410774992E-04
K8	-4.996497300533E-01	4.991175267737E-01	4.991175267737E-01
TA9	2.528143928200E-01	2.515113889155E-01	2.515113889155E-01
NB10	2.528416287669E-01	2.515953433859E-01	-2.484199794154E-01
NB11	2.528416287669E-01	-2.484199794154E-01	2.515953433859E-01
TA12	2.528421307941E-01	-2.484725195252E-01	-2.484725195252E-01
NB13	-2.471162030615E-01	2.515664298828E-01	2.515664298828E-01
TA14	-2.472278432257E-01	2.515228575329E-01	-2.485003942727E-01
TA15	-2.472278432257E-01	-2.485003942727E-01	2.515228575329E-01
NB16	-2.470846112526E-01	-2.484090651371E-01	-2.484090651371E-01
O17	-1.206896377477E-03	2.458519264711E-01	2.458519264711E-01
O18	-3.971111108313E-03	2.458370500183E-01	-2.541354412170E-01
O19	-3.971111108313E-03	-2.541354412170E-01	2.458370500183E-01
O20	-1.204248182983E-03	-2.541863195163E-01	-2.541863195163E-01
O21	4.960307242820E-01	2.458548298477E-01	2.458548298477E-01
O22	4.987912187111E-01	2.458115691297E-01	-2.541380164971E-01
O23	4.987912187111E-01	-2.541380164971E-01	2.458115691297E-01
O24	4.960332460713E-01	-2.541562683831E-01	-2.541562683831E-01
O25	2.469873761848E-01	-2.348134726299E-03	2.458355726933E-01
O26	2.469293787612E-01	-5.121413342747E-03	-2.541881753529E-01
O27	2.469432316939E-01	4.948897023733E-01	2.458138241615E-01
O28	2.470028481742E-01	4.976400070523E-01	-2.541743400842E-01
O29	-2.530530766870E-01	-5.110731837940E-03	2.458118313945E-01
O30	-2.529939501449E-01	-2.363362174401E-03	-2.541752827323E-01
O31	-2.530062576473E-01	4.976495913164E-01	2.458329270237E-01

```
O32  -2.530685281582E-01  4.948766673830E-01 -2.541913616142E-01

O33   2.469873761848E-01  2.458355726933E-01 -2.348134726299E-03

O34   2.469432316939E-01  2.458138241615E-01  4.948897023733E-01

O35   2.469293787612E-01 -2.541881753529E-01 -5.121413342747E-03

O36   2.470028481742E-01 -2.541743400842E-01  4.976400070523E-01

O37  -2.530530766870E-01  2.458118313945E-01 -5.110731837940E-03

O38  -2.530062576473E-01  2.458329270237E-01  4.976495913164E-01

O39  -2.529939501449E-01 -2.541752827323E-01 -2.363362174401E-03

O40  -2.530685281582E-01 -2.541913616142E-01  4.948766673830E-01


#===END
```

2 SI Figures

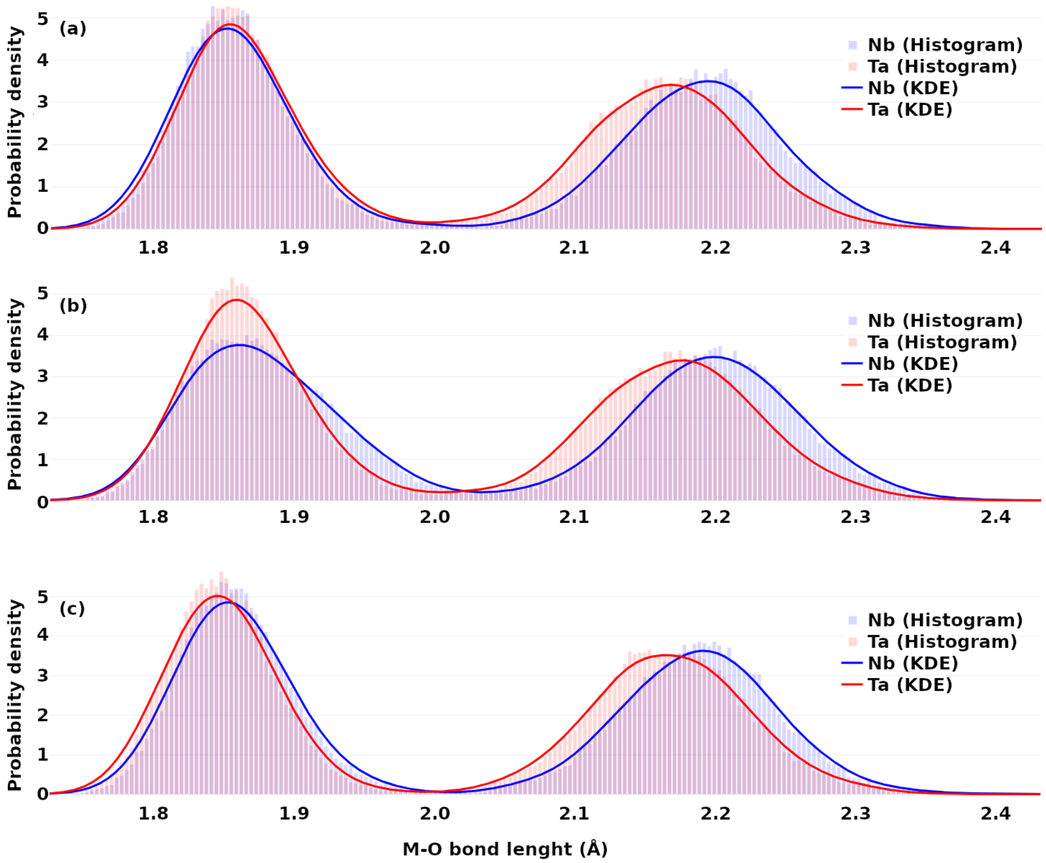

Figure SI-1: Probability density distributions of M–O bond lengths (M = Nb or Ta) obtained from ab initio molecular dynamics (AIMD) simulations for the three structural models of $KTa_{0.5}Nb_{0.5}O_3$: (a) $[100]_{\mathrm{NT}}$, (b) $[101]_{\mathrm{NT}}$, and (c) $[111]_{\mathrm{NT}}$. Histograms represent the raw bond length distributions, while the solid lines correspond to kernel density estimates (KDE) for Nb–O (blue) and Ta–O (red) interactions. The bimodal behavior highlights the coexistence of short and long M–O bonds in all configurations, with Ta–O bonds generally exhibiting shorter average lengths than Nb–O, particularly in the $[101]_{\mathrm{NT}}$ model.
